# A case study using the United Republic of Tanzania: costing nationwide HPV vaccine delivery using the WHO Cervical Cancer Prevention and Control Costing Tool

**DOI:** 10.1186/1741-7015-10-136

**Published:** 2012-11-13

**Authors:** Raymond Hutubessy, Ann Levin, Susan Wang, Winthrop Morgan, Mariam Ally, Theopista John, Nathalie Broutet

**Affiliations:** 1 Immunization, Vaccines and Biologicals (IVB) Department, World Health Organization (WHO), 20 Avenue Appia, 1211, Geneva 27, Switzerland; 2 Independent consultant, 6414 Hollins Dr., Bethesda, MD, 20817, USA; 3 Ministry of Health and Social Welfare (MOHSW), PO Box 9083, Dar es Salaam, United Republic of Tanzania; 4 WHO Country Office, PO Box 9292, Dar es Salaam, United Republic of Tanzania; 5 Reproductive Health and Research (RHR) Department, WHO, 20 Avenue Appia, 1211, Geneva 27, Switzerland

**Keywords:** Human papillomavirus (HPV), vaccines, immunization programs, costing, planning, United Republic of Tanzania, low- and middle-income countries, GAVI Alliance, GAVI eligible countries

## Abstract

**Background:**

The purpose, methods, data sources and assumptions behind the World Health Organization (WHO) Cervical Cancer Prevention and Control Costing (C4P) tool that was developed to assist low- and middle-income countries (LMICs) with planning and costing their nationwide human papillomavirus (HPV) vaccination program are presented. Tanzania is presented as a case study where the WHO C4P tool was used to cost and plan the roll-out of HPV vaccines nationwide as part of the national comprehensive cervical cancer prevention and control strategy.

**Methods:**

The WHO C4P tool focuses on estimating the incremental costs to the health system of vaccinating adolescent girls through school-, health facility- and/or outreach-based strategies. No costs to the user (school girls, parents or caregivers) are included. Both financial (or costs to the Ministry of Health) and economic costs are estimated. The cost components for service delivery include training, vaccination (health personnel time and transport, stationery for tally sheets and vaccination cards, and so on), social mobilization/IEC (information, education and communication), supervision, and monitoring and evaluation (M&E). The costs of all the resources used for HPV vaccination are totaled and shown with and without the estimated cost of the vaccine. The total cost is also divided by the number of doses administered and number of fully immunized girls (FIGs) to estimate the cost per dose and cost per FIG.

**Results:**

Over five years (2011 to 2015), the cost of establishing an HPV vaccine program that delivers three doses of vaccine to girls at schools via phased national introduction (three regions in year 1, ten regions in year 2 and all 26 regions in years 3 to 5) in Tanzania is estimated to be US$9.2 million (excluding vaccine costs) and US$31.5 million (with vaccine) assuming a vaccine price of US$5 (GAVI 2011, formerly the Global Alliance for Vaccines and Immunizations). This is equivalent to a financial cost of US$5.77 per FIG, excluding the vaccine cost. The most important costs of service delivery are social mobilization/IEC and service delivery operational costs.

**Conclusions:**

When countries expand their immunization schedules with new vaccines such as the HPV vaccine, they face initial costs to fund critical pre-introduction activities, as well as incremental system costs to deliver the vaccines on an ongoing basis. In anticipation, governments need to plan ahead for non-vaccine costs so they will be financed adequately. Existing human resources need to be re-allocated or new staff need to be recruited for the program to be implemented successfully in a sustainable and long-term manner.

Reaching a target group not routinely served by national immunization programs previously with three doses of vaccine requires new delivery strategies, more transport of vaccines and health workers and more intensive IEC activities leading to new delivery costs for the immunization program that are greater than the costs incurred when a new infant vaccine is added to the existing infant immunization schedule. The WHO C4P tool is intended to help LMICs to plan ahead and estimate the programmatic and operational costs of HPV vaccination.

## Background

Cervical cancer caused by infection with carcinogenic types of human papillomavirus (HPV) is the second most common cancer in women worldwide according to age-standardized incidence rates (ASR). In 2008, there were more than a half million new cases and 274,000 deaths due to cervical cancer [1]. More than 85% of these cases occurred in low- and middle-income countries (LMICs), with the highest incidence rates in Sub-Saharan Africa, South-Central Asia, Latin America and Melanesia [2]. The United Republic of Tanzania has one of the highest cervical cancer burdens in the world and the highest in Eastern Africa, with an ASR of 50.9 cases per 100,000 women.

The World Health Organization (WHO) recommends routine vaccination of 9- to 13-year-old girls to protect against HPV infections with types 16 and 18, which contribute to the development of approximately 70% of cervical cancers [3] in countries where: (1) the prevention of cervical cancer and/or other HPV-related diseases is a public health priority; (2) vaccine introduction is programmatically feasible; (3) sustainable financing can be secured; and (4) the cost-effectiveness of vaccination strategies in the country or region has been duly considered [3]. In most LMICs, the vaccine price offered to the public sector (ranging from US$15 to more than US$130 per dose) has been a barrier for vaccine uptake. The GAVI Alliance (formerly the Global Alliance for Vaccines and Immunization) is opening a finance window to purchase HPV vaccine at or about US$5 per dose in order to co-finance the vaccine with GAVI-eligible countries that apply and receive approval [4]. While the vaccine price is a key driver on the cost side, national governments and donors will also need to consider additional resources to support implementation costs associated with delivery of vaccine to a new target population that has not been routinely vaccinated previously. To date, while vaccine delivery costs for small-scale demonstration projects in India, Peru, Uganda, Vietnam [5] and Tanzania [6] have been reported, data are lacking on large-scale, country-wide implementation costs. Hence, information on the affordability of different HPV vaccine delivery strategies in LMICs is limited.

As part of developing Tanzania's national comprehensive strategy for cervical cancer prevention and control, the Ministry of Health and Social Welfare (MOHSW) requested technical assistance from WHO to estimate the service delivery costs of introducing HPV vaccine into the country. This request was also in response to a MOHSW agreement to accept a three-year donation of HPV vaccine from the manufacturer. To address this request, WHO and their consultants developed the Cervical Cancer Prevention and Control Costing (C4P) tool in order to facilitate the decision-making process of program managers and policy makers by generating information on the projected costs of introducing cervical cancer interventions. The WHO C4P tool consists of a HPV Vaccine Module and a Cervical Cancer Screening and Treatment Module. As the latter component is still under development, this paper will focus only on the HPV vaccination component of the tool.

This paper aims to present the purpose, definitions, methods, data sources and assumptions behind the generic WHO C4P tool to assist LMICs with planning and costing their nationwide HPV vaccination program. Furthermore, Tanzania's experience and cost results will be presented in piloting the WHO C4P tool to scale up prevention interventions as part of their national comprehensive cervical cancer prevention and control plan.

## Methods

### Purpose of the generic tool

The generic costing tool is a country-specific costing and planning tool that facilitates data collection and enables the user to estimate and project the value of incremental (additional) resources required to add the country-wide delivery of HPV vaccine to an existing immunization program over a five-year period. In other words, it only estimates the value of new resources needed and does not include the cost of other goods and services (for example, transport) already being used for other vaccines (shared costs). For example, it does not estimate the cost of transporting HPV vaccine if this is part of the same transport used to deliver other vaccines such as rotavirus or traditional Expanded Program on Immunization (EPI) vaccines, such as diphtheria, tetanus and pertussis (DTP) from the central warehouse to the periphery in the country. Since the WHO C4P tool focuses on estimating the incremental costs of vaccinating adolescent girls from a public health care provider perspective, no costs to the user (school girls, parents and caregivers) are included.

Experience from the above-mentioned small-scale demonstration projects showed [5,6] that the quantity of resources required to introduce HPV vaccine for national immunization programs (NIPs) will differ from those for new infant vaccines since it has a non-traditional target population--annual cohorts of girls 9- to 13-years old. Reaching this new and older target population with three doses of vaccine requires new delivery infrastructure, more transport for vaccines and health workers and more intensive IEC activities, leading to higher costs per vaccinated girl than costs per vaccinated infant for a new infant vaccine. That is, since the vaccine may be administered at venues such as schools or places in the community additional costs are incurred for outreach.

The HPV Vaccine Module of the WHO C4P tool enables the user to estimate the additional resource requirements based on identifying different vaccine delivery scenarios that could potentially be considered for the country. The tool allows the user to define different vaccine delivery strategies, for example, through schools, health facilities or campaigns, such as national immunization days. The tool provides estimates of several cost measures: (1) total costs of introducing the HPV vaccine in specific regions/provinces or at the national level; (2) delivery cost per dose; and (3) delivery cost per fully immunized girl (FIG) defined as the cost per dose multiplied by the total number of doses delivered over three vaccination rounds divided by the total number of girls who received all three doses as a function of coverage and dropout rates over all three vaccination rounds.

**Figure 1:**
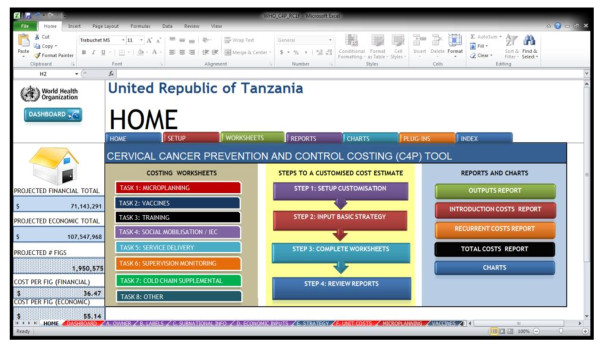
**Screenshot of C4P Tool**.

The generic version of the WHO C4P tool is built on the experience of Tanzania. It was further developed and critically assessed by costing experts representing five WHO regions during a WHO workshop in December 2011 in Geneva. A beta version of the MS Excel tool (see Figure 1 for a screenshot), including a User Guide, is accessible through the WHO New and Under-Utilized Vaccines Implementation (NUVI) website [7].

### Cost categories and components of the tool

The WHO C4P tool allows the user to estimate the costs of activities that take place during the introduction of HPV vaccination into a national immunization program. These activities include the following: procurement of vaccines and injection supplies, micro-planning, training, social mobilization and IEC, purchase of cold chain equipment, service delivery of vaccines to target population, monitoring and evaluation, supervision and waste management. Typical users of the WHO C4P tool are national health planners without prior experience of HPV vaccine introduction.

The tool differentiates recurrent (operational) and capital costs as well as financial and economic costs. It also presents expenditures required for investments necessary during the first years of HPV vaccine introduction.

Recurrent costs are defined as the value of resources that last less than one year. These include program costs such as the value of personnel time, transport, maintenance, monitoring and evaluation, and supervision as well as costs of short-term training activities that last less than a year (for example, do not include material development and initial training).

Capital costs are the value of resources that last longer than one year, such as cold chain equipment and vehicles. The capital goods and services used in HPV vaccination include initial investments such as introduction costs (micro-planning, initial training and social mobilization/IEC material development) as well as additional cold chain equipment, vehicle requirements and incinerators.

Both financial and economic costs are calculated in the WHO C4P tool. The user can choose which one is most appropriate depending on the objective of the analysis. If the user wants to know the additional costs incurred or actual expenditures by the Ministry of Health, for example, they should focus on the financial cost calculation. Financial costs are the value of resources to the payer and include the value of actual resources purchased for the HPV vaccine introduction such as injection supplies, outreach allowances and per diem, resources used in training, and developing new communication materials useful for budgeting purposes.

Economic costs comprise the value of all outlays for vaccine introduction as well as those already paid for or owned by the Ministry of Health and other sources of financing, for example, the salaries of health personnel, vaccines paid for by partners and time of volunteers. This analysis is useful if the user is interested in evaluating the share of different sources of finance for the vaccine introduction. For example, they may want to know the share of total costs financed by the Ministry of Health, external partners, clients and the community. This analysis gives a more complete picture of resources that are tied up in the provision of the new vaccine and their opportunity costs and should be used if a cost-effectiveness or cost-benefit analysis is to be conducted.

Capital costs are calculated differently depending on whether financial or economic costs are being estimated. When calculating financial costs, straight-line depreciation is used in the calculation of capital costs. In other words, the cost of the item is annualized through dividing it by the useful life years of the good. For example, cold chain equipment could be expected to last for ten years and the total cost would be divided by ten. Straight-line depreciation assumes that capital goods are used up equally over the useful time period of the item. For economic costs, capital goods are discounted (at a 3% default discount rate) as well as annualized. This type of depreciation assumes that people have time preference and prefer to use goods and services now rather than in the future.

The main differences between financial and economic costing are: (1) the time spent by health personnel, school teachers and volunteers is valued in economic costing since there is an opportunity cost to this time - for example, the workers are unable to spend time on other activities when they are occupied with HPV vaccination - but are not included in financial costs since these are already paid for with government salaries; (2) the value of donated goods and services is included in economic costs but not in financial costs since there is an opportunity cost to their use; and (3) capital costs are calculated differently for financial and economist costs.

For planning purposes the user is able to identify and separate introduction costs (treated as a type of capital or fixed costs) from recurrent costs. Introduction costs are defined as resources required during the initial years of vaccine introduction (some may occur during the second or third year if the country has phased in vaccine introduction) while the recurrent costs are the running costs of the program such as transport of vaccines and health workers, allowances and per diems and monitoring and supervision.

### Data sources and other assumptions of WHO C4P tool

During October 2010 to May 2011, data were collected from the MOHSW/EPI on the costs of training, social mobilization/IEC, vaccination, supervision, and monitoring and evaluation. The study team collected primary and secondary data on target populations, health facilities, schools and costs of resources used to provide HPV vaccination from the MOHSW website, and Ministry of Education website (see also Table 1). The team also interviewed MOHSW/EPI program managers and other partners (WHO and UNICEF) to obtain information on programmatic options and cost data. In addition, they supplemented these data with a survey on unit costs of hall rentals, per diems, travel allowances and production costs for IEC materials and training manuals. Furthermore, United Nations Development Program (UNDP) population data and the Tanzanian Comprehensive Multi-Year Plan (cMYP) were used. Information was also collected from the London School of Hygiene and Tropical Medicine (LSHTM)/National Institute of Medical Research pilot project of school-based introduction of HPV vaccination in Mwanza province [6].

**Table 1:** Data Sources for Tanzanian Cost Analysis of HPV Vaccine Introduction.

Data	Source
Number of 10 year old girls	UNDP
Number of health facilities, types and number of health staff	MOHSW
Number of primary schools	Ministry of Education
Health Staff Salaries	cMYP
Unit Costs of hall rentals, facilitator fees, and other meetingexpenses, production of IEC materials^a^	Survey of local costs
Unit costs of supplies	MOHSW, WHO
Transport allowances and per diems	MOHSW, WHO
Exchange and inflation rates	Ministry of Finance
Vaccine cost	Estimate from GAVI Alliance 2011

^a^The unit cost of hall rentals, for example, includes the cost per day per hall rental for a meeting or training. This unit cost is multiplied by the number of days of a meeting or training to get the cost of hall rental per training or meeting. cMYP, Comprehensive Multi-Year Plan (Tanzania); GAVI, Global Alliance for Vaccines and Immunization; MOHSW, Ministry of Health and Social Welfare; UNDP, United Nations Development Plan; WHO, World Health Organization.

The data for the Tanzania analysis were taken from several information sources, as can be seen in Table 1, ranging from the MOHSW, the Ministry of Education (MOE) and the Ministry of Finance (MoF). The population estimates of ten-year-old girls were taken from UNDP.

### The WHO C4P Vaccine Module: A case study for Tanzania

An overview of the basic assumptions of the HPV vaccination program in Tanzania is summarized in Table 2. It should be highlighted that, in this analysis, Tanzania assumed that no additional costs for cold chain would be required for HPV vaccine introduction.

**Table 2:** Basic assumptions for HPV vaccination program in Tanzania.

Number	Description of assumption
1.	Vaccine will be phased in over three years: 3 regions the first year, 10 regions thesecond year, and all 26 regions the 3^rd^, 4^th ^and 5^th ^years.
2.	Girls enrolled in Primary 4 are a proxy of 10-year-old girls.
3.	Four visits will be made to each school for orientation and to reach all of the girls.
4.	No additional costs for the cold chain will be required.
5.	Transport for bringing the vaccine to the health facilities will be integrated into existingtransport for EPI vaccines.
6.	The coverage for girls age 10 is 85%, 77% and 65% in the 1^st^, 2^nd ^and 3^rd ^roundrespectively, vaccine wastage is 5%, a buffer stock of 25% is maintained.
7.	The price per dose is assumed to be US$5 per dose based on the price offered to theGAVI Alliance by Merck.
8.	The health worker spends half a day at each school and receives outreach per diem
9.	The transport allowance from the health facility for a health worker to and from theschool costs on average 10,000 Tsh (US$6.30)
10.	Vaccines will be donated during the first three years but MOHSW will pay for syringes,receiving, clearance, storage and transport of the vaccines to the health facilities.

EPI, Expanded Program on Immunization; GAVI, Global Alliance for Vaccines and Immunization; MOHSW, Ministry of Health and Social Welfare.

### Proposed HPV vaccine delivery strategy in Tanzania

The MOHSW of Tanzania is planning a phased school-based delivery strategy, beginning in three regions of the country, expanding to ten regions in the second year, and covering 26 regions during the third through fifth years. Nurses from health centers and dispensaries will travel to schools to provide vaccinations to girls in the Primary 4 level in the schools. The health workers will visit each school three to four times for planning and administration of three vaccine doses to the girls.

In preparation for the vaccination, the MOHSW will conduct trainings for health staff and develop and undertake sensitization meetings with communities, parents and the media to inform the general population about the benefits of the vaccination through IEC.

### Planned activities in Tanzania

Training activities that are costed include a curriculum development workshop and the following trainings: training of trainers (ToT) workshops, district supervisors and vaccinators. Social mobilization and IEC activities included the following: (1) a sensitization, mobilization and communication guidelines and tools preparation workshop; (2) a district campaign preparation workshop; (3) materials development and production; (4) materials printing and airing; (5) sensitization meetings with the community; and (6) a media seminar for journalists. Radio spots are projected to air all three years, but national television advertisements will only take place in the third year when the vaccination is introduced in all of the regions, including Zanzibar.

The financial costs of service delivery are comprised of per diems and transport allowances for the health worker and per diems for the school teacher which have to be paid on top of their regular salaries. Procurement costs are incurred for receiving and storage of the vaccines at the port and transport of the vaccines to the zonal level, but do not include vaccine costs since these are donated commodities. The monitoring and evaluation costs are comprised of production costs for tally sheets and vaccination cards. The costs of supervision are for transport (fuel and maintenance of vehicles) and per diems incurred during the quarterly visits from the national level to the regions and districts. The waste management costs are for construction of incinerators, fuel for incinerators and/or transport of waste.

Economic costs of service delivery include the value of resources for financial costs as well as the value of the time of personnel time and donated vaccines and capital items.

## Results

In the scenario examined, 1.6 million girls are fully vaccinated (that is, receive three doses) over five years out of a total target population of 2.4 million girls (five cohorts of ten-year-old girls in the five-year national program with phased roll-out during years one to two and national vaccination starting in year three for an estimated three-dose vaccine coverage of 65%.) Table 3 provides a summary of the expected outputs of introducing HPV vaccines in Tanzania.

**Table 3:** Summary of expected outputs of introducing HPV vaccines in Tanzania, 2011-2015.

Output	2011	2012	2013	2014	2015	Total
Target population	80,290	349,349	646,936	672,833	694,666	2,444,074
Doses used^a^	181,878	791,362	1,471,308	1,524,135	1,573,591	5,542,274
Fully immunized girls	52,209	227,164	422,345	437,510	451,706	1,590,934
Health workers trained	1,472	3,596	5,070	0	0	10,138
Health facilities mobilized	736	1,798	2,535	0	0	5,069
School vaccination sites added	2,098	6,333	7,727	0	0	16,158
Schools sensitized	2,098	6,333	7,727	0	0	16,158

^a^Doses used include doses administered, wastage and buffer. HPV, human papillomavirus.

The five-year cost of introducing a national phased-in HPV vaccine program during 2011 to 2015 in Tanzania is estimated to be US$9.2 million (excluding vaccine costs) and US$31.5 million (with vaccine) assuming a vaccine price of US$5 (GAVI 2011). This is equivalent to a financial cost of US$5.77 per fully immunized girl, excluding the vaccine cost.

**Figure 2:**
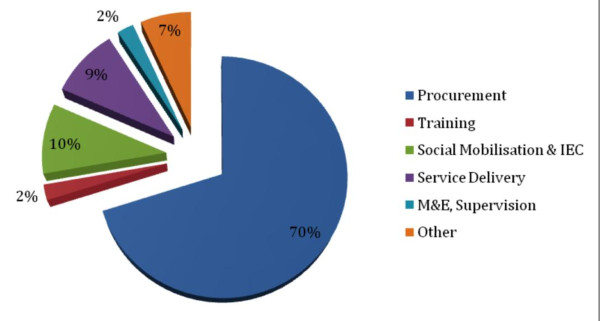
**Financial costs of HPV vaccine delivery, 2011 to 2015**.

Table 4 shows the breakdown of costs during the first five years of the vaccine introduction by activities which totals to US$31.5 million (with vaccines). Excluding procurement, the largest share of costs goes towards social mobilization and IEC, followed by service delivery. Figure 2 shows a pie chart of the financial costs breakdown by activities of introducing HPV vaccine in Tanzania during 2011 to 2015. These data show that substantial funding is required for HPV vaccine delivery efforts despite the fact the vaccine will be donated during the first three years.

**Table 4:** Financial Costs of Introducing HPV Vaccine in Tanzania, 2011-2015 (2011 US$).

Activity/Year Number of regions	2011 3 regions	2012 10 regions	2013 26 regions	2014 26 regions	2015 26 regions	Total
Procurement^a^	132,880	552,459	963,346	10,162,160	10,491,906	22,302,751
Training ^b^	140,489	203,783	298,912	-	-	643,184
Social mobilization and IEC	191,431	472,009	941,007	661,853	668,039	2,934,340
Service Delivery^c^	97,907	393,447	754,040	754,040	754,040	2,753,473
M and E, Supervision^d^	50,181	97,191	171,731	171,731	171,731	662,564
Other^e^	72,972	312,303	583,713	602,214	619,534	2,190,735
Totals	685,860	2,031,190	3,712,749	12,351,998	12,705,250	31,487,047

^a^Procurement includes the cost of vaccines, including shipping, receiving and storage, plus 5% wastage, and buffer stock; ^b^training includes costs of transport to the meeting venues, supplies, facilitators, hall rentals, and personnel allowances and lodging; ^c^service delivery includes transport to schools, outreach per diems/allowances for health workers and school teachers, and health workers and school teacher time (for economic costs); ^d^monitoring and evaluation and supervision include monitoring forms, vaccination cards, transport for supervisory visits, and health worker personnel allowances and lodging; ^e^other includes fuel and transport for waste management. IEC, information, education and communication; M and E, management and evaluation.

**Figure 3:**
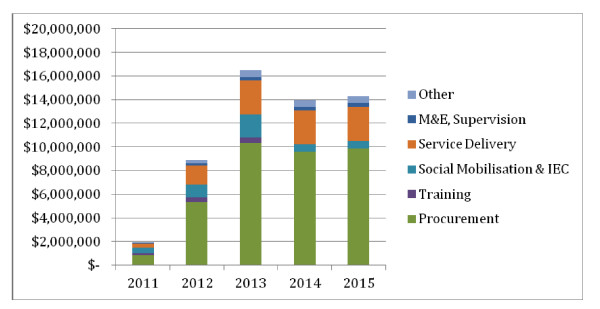
**Economic costs of HPV Vaccine, 2011 to 2015**.

Figure 3 shows the economic costs of HPV vaccine by cost component. The resource requirements over five years that include shared costs (for example, transport or salaries also used for other vaccines) are approximately US$58 million.

Table 5 shows the financial and economic costs per dose and FIG without and with vaccines during 2011 to 2015 in terms of financial and economic costs. Without the vaccine costs the financial costs per dose and per FIG are US$1.66 and US$5.77, respectively, while the economic costs (including salary costs of MOHSW personnel) per dose and per FIG are US$3.56 and US$12.40. The average cost per dose and FIG are higher if the vaccine costs are included, as shown in Table 5.

**Table 5:** Financial and economic cost per dose and per fully immunized girl without and with vaccine costs (2011 US$).

	**Without vaccine**		**With vaccine**	
	
	**Financial Cost**	**Economic Cost**	**Financial Cost**	**Economic Cost**
	
Cost per dose	1.66	3.56	5.68	10.62
Cost per FIG^a^	5.77	12.40	19.79	37.01

^a^The coverage of FIG is 65% assuming coverage rates for girls age 10 of 85%, 77% and 65% in the 1^st^, 2^nd ^and 3^rd ^round, respectively. FIG, fully immunized girl.

Table 6 shows an illustrative comparison using the WHO C4P tool of school-based and health facility-based scenarios in terms of financial introduction and recurrent costs per eligible girl. The introduction costs during the first three years of phased roll-out would be similar; however, the recurrent costs per dose and for three doses per eligible girl would be higher for a school-based compared to health facility-based delivery scenario because transportation costs of vaccines and personnel per diems will be higher for the outreach.

**Table 6:** Financial Costs of Introducing HPV Vaccine through school- and health facility-based strategies in Tanzania, 2011-2015 (2011 US$).

	Delivery strategy: School-based	Delivery strategy: Health facility- based
Age of target population Number of girls in target population	10 years 605, 000	10 years 605, 000
Introduction costs per eligible girl	3.07	3.07
Recurrent cost per dose	1.59	1.17
Recurrent costs for three doses per eligible girl	4.78	3.51

## Discussion

When countries expand their immunization schedule with a new vaccine such as HPV, they face initial costs to fund critical pre-introduction activities, as well as incremental system costs to deliver the vaccines on an ongoing basis. By using the WHO C4P, the Tanzania experience of introducing HPV vaccine through a phased school-based delivery strategy in all the 26 regions shows that the five-year recurrent total costs of introducing HPV vaccine (excluding vaccine cost) are around US$9.2 million, which is equivalent to US$5.77 per FIG to the government. These recurrent costs correspond to 0.7% of the government real expenditures on health, indicating that substantial ministry of health government resources, such as health personnel, are required to deliver the vaccine effectively. Actual resources required are dramatically higher when one considers the vaccine costs and shared costs included as part of the economic costs analysis, that is, approximately US$59 million over a five-year period.

This study shows that, other than procurement, the most important costs of service delivery are social mobilization/IEC and service delivery operational costs. Social mobilization and IEC activities are particularly important to ensure that broad coverage is achieved by informing the population about the benefits of HPV vaccination and supporting the service delivery strategy used. Governments need to plan ahead for these non-vaccine costs so that they will be financed adequately and human resources need to be re-allocated appropriately for the program to be successfully implemented in a sustainable and long-term manner.

To date, data availability on national HPV vaccination in LMICs is limited, which makes it difficult to validate the tool. However, the findings from the WHO C4P tool for Tanzania are consistent with existing information on recurrent costs from various small-scale demonstration projects from the Program for Appropriate Technology in Health (PATH) in India, Peru, Uganda and Vietnam [5] and the LSHTM project in Mwanza province in Tanzania [6]. Furthermore, the WHO C4P tool for Tanzania findings are also consistent with scale-up cost estimates for national HPV vaccination studies using the WHO C4P tool for Uganda [6], Bhutan and Rwanda based on actual HPV vaccine introduction with actual expenditure data (personal communication with responsible EPI officers from Bhutan and Rwanda)^a^. These studies demonstrate that introduction costs for HPV vaccines are higher than those for existing vaccines such as meningitis A campaigns and combination DTP-HepB-Hib vaccine via routine infant EPI vaccination programs [8,9] due to increased needs for IEC and social mobilization activities to sensitize the public about the benefits of HPV vaccines for adolescent girls.

From the nationwide-modeled experiences based on PATH demonstration projects, the average introduction costs per eligible girl are US$2.99 (range US$2.82 to US$3.07) and the recurrent costs to deliver three doses per eligible girl are US$4.17 (range US$3.51 to US$4.78) [5]. As HPV vaccine introduction requires building up a new delivery infrastructure, the costs are significantly higher. From the three available projected nationwide-modeled HPV vaccination costing studies in Tanzania, Uganda and Bhutan, the average cost is about US$3.00 per 10-year old girl.

The resource requirements of IEC activities are a large component of total costs since these are considered to be important aspects for a successful introduction of HPV vaccination, a new vaccine that targets a non-traditional population of adolescent girls. The population will need to be assured of its safety and benefits and be provided with an explanation of why this vaccination is given only to girls and not boys. The costs of IEC activities are calculated for the following activities: (1) sensitization meetings with community leaders to inform them of the benefits of the intended vaccination activities; (2) production of leaflets and posters on the benefits of HPV vaccinations to be placed by service providers in clinics, schools and public locations in their catchment areas; (3) design and production of radio and/or television announcements on the HPV vaccine for the population; and (4) briefings with writers, journalists, editors, publishers and other media personnel to inform them about the benefits of the vaccine. As HPV vaccination is scaled up in these countries, more IEC activities will be required, given that airing of radio and TV announcements will be more effective once the vaccination is scaled up nationally.

The WHO C4P tool in its current version has several limitations. First, depending on the countries' characteristics, additional sizable costs might be expected from a societal perspective that are not included in the WHO C4P tool, such as private costs to schoolgirls, parents and caregivers and additional costs for the cold chain. Variation in the incremental cost to the health system of vaccinating adolescent girls by different countries is expected and can potentially be explained by country characteristics, such as size of the country, population density and proximity of health facilities to schools, current infrastructure of schools and health facilities and national income level as well as the intensity of the HPV vaccine introduction effort (Levin *et al*. in preparation). Secondly, monitoring and evaluation costs are restricted to production of tally sheets and vaccination cards. In reality, additional quality control or evaluative measures, such as cost of administrative personnel to evaluate coverage levels might be required.

Past experience from the African region for delivery of other adolescent health interventions such as school deworming programs with benzimidazoles [10] and school-targeted treatment for *Schistosoma mansoni *[11] suggest lower cost estimates per child compared to delivering an adolescent vaccine. More recently, costing data is becoming available on the delivery and scaling up of sexual and reproductive health interventions through adolescent-friendly health services. Pilot testing of a WHO costing tool in Uganda in 2006 found unit costs per adolescent child ranging from US$4.50 for sexually transmitted infection (STI) treatment in a public primary health facility to US$19 for HIV counseling and testing in a non-governmental organization (NGO)-run facility [12]. Overall, however, accurate cost estimates of interventions delivered to young people are rare and more needs to be done to improve evaluations of the economic value of investments targeted at this age group [13].

In anticipation of these additional service delivery costs for HPV vaccines as an example for non-traditional vaccines, the GAVI Alliance is reviewing its current policy towards vaccine introduction grants and operational support for campaigns [14]. GAVI's introduction grant is a one-time cash grant to fund some of the activities associated with adopting a new vaccine in a country's national immunization program [15]. However, the vaccine introduction grant does not fund the total costs resulting from a new vaccine introduction. Governments and partners are expected to contribute additional funding in order to facilitate an effective introduction. Past experience has shown that governments have not always been able to quickly mobilize additional funding from their own budgets or development partners to fill these funding gaps. The WHO C4P tool could be used to assist countries in estimating the pre-introduction and incremental system costs to deliver the HPV vaccine.

Use of an early version of the Cervical Cancer Screening and Treatment Module of the WHO C4P tool demonstrates that the preliminary costs of scaling-up screening and treatment in Tanzania are estimated to be $12.1 million over five years. In the scenario examined, 1.2 million out of a target population of five million women are screened using visual inspection with acetic acid (VIA), while 17.4 thousand women out of 60 thousand VIA-positive women receive treatment. This estimate is based on screening at the health dispensary level and above and treatment at the health center level and above [16]. Completion of the Cervical Cancer Screening and Treatment Module of the WHO C4P tool will further contribute to the decision-making process.

Finally, HPV vaccines are not the only new vaccines being considered for introduction in LMICs. There are also pneumococcal and rotavirus vaccines as well as several older vaccines that have not yet been broadly adopted in developing countries. Although intended for national HPV vaccination costing and planning purposes, the economic cost results of the WHO C4P tool can be used as an input for cost-effectiveness analysis and/or budget impact analysis in order to assist countries in setting their priorities between competing vaccines or other cervical cancer control options. In-country cost estimates of the programmatic costs of delivering an adolescent vaccine and scaling up of cervical cancer screening and treatment interventions as an input for cost-effectiveness analysis are rare. For instance, a health and economic impact study of HPV vaccination and cervical cancer screening in five Eastern African countries would have benefited from a country-specific data collection and projection costing tool such as WHO C4P to estimate these programmatic costs [17] among other tools.

## Conclusions

The financial delivery costs of nationwide HPV vaccination are higher than those of infant vaccines and can be substantial in resource-poor settings since it requires building up new delivery channels. As a consequence, governments need to plan ahead for these non-vaccine costs so that they will have adequate finances in place for vaccine introduction.

As GAVI's vaccine portfolio is expanding and countries are expected to introduce new vaccines at an increasing rate, it is recommended to include (partial) funding for operational health system costs in order assist GAVI eligible countries in introducing HPV vaccines. Together with other decision-making tools, the WHO C4P tool could facilitate both low- and middle-income countries in demonstrating their ability to deliver HPV vaccines nationwide to the target population in an effective, sustainable and affordable manner.

## Abbreviations

ASR: age-standardized incidence rates; C4P: cervical cancer prevention and control costing; CEA: cost-effectiveness analysis; cMYP: comprehensive multi-year plan; EPI: Expanded Program on Immunization; FIG: fully immunized girl; GAVI Alliance: formerly the Global Alliance for Vaccines and Immunisation; HPV: human papillomavirus; IEC: information: education and communication; LMICs: low- and middle-income countries; LSHTM: London School of Hygiene and Tropical Medicine; M&E: monitoring and evaluation; MOHSW: Ministry of Health and Social Welfare; NIP: national immunization program; NUVI: new and under-utilized vaccines implementation; PATH: Program for Appropriate Technology in Health; UNDP: United Nations Development Program; VIA: visual inspection with acetic acid; WHO: World Health Organization.

## Competing interests

The authors declare that they have no competing interests.

## Authors' contributions

RH, AL, SW and NB contributed to the conception and design of the study and the C4P tool. AL and WM developed the C4P tool under supervision and directions from RH, SW and NB. RH, SW, AL, MA and TJ were responsible for the acquisition of data in Tanzania. RH, SW, AL, WM and MA analyzed and interpreted the data; all co-authors have been involved in drafting the manuscript or revising it critically for important intellectual content. All authors have read and approved the final manuscript.

## End Notes

^a^Personal communication with Mr. Tandin Dorji, Chief Program Officer, Department of Public Health, Ministry of Health, Bhutan; and Mr. Maurice Gatera, Head of Vaccine Preventable Disease Division, Institute of HIV/AIDS Disease Prevention and Control, Rwanda Biomedical Center, Ministry of Health, Rwanda. Permission was obtained to quote their names.

## Pre-publication history

The pre-publication history for this paper can be accessed here:

http://www.biomedcentral.com/1741-7015/10/136/prepub
